# Changes in CHA_2_DS_2_-VASc score and risk of ischemic stroke among patients with atrial fibrillation

**DOI:** 10.1007/s00380-023-02278-1

**Published:** 2023-06-13

**Authors:** Eirinaios Tsiartas, Athanasios Samaras, Andreas S. Papazoglou, Anastasios Kartas, Dimitrios V. Moysidis, Eleftherios Gemousakakis, Odysseas Kamzolas, Alexandra Bekiaridou, Ioannis Doundoulakis, Apostolos Tzikas, George Giannakoulas

**Affiliations:** First Cardiology Department, School of Medicine, Faculty of Health Sciences, AHEPA University Hospital, Aristotle University of Thessaloniki, 1 Kiriakidi, Thessaloniki, 546 36 Greece

**Keywords:** Atrial fibrillation, Ischemic stroke, Oral anticoagulation, CHA2DS2-VASc score, Stroke risk

## Abstract

**Aims:**

The CHA_2_DS_2_-VASc score is fundamental to stroke risk assessment in atrial fibrillation. However, stroke-related risk factors can be modified later in life. This study aimed to assess the association of changes in CHA_2_DS_2_-VASc score over time (Delta CHA_2_DS_2_-VASc score) with the risk of ischemic stroke.

**Materials and methods:**

This is an observational analysis of 1127 atrial fibrillation patients previously enrolled in the MISOAC-AF trial. After a median 2.6-year follow-up period, baseline and follow-up CHA_2_DS_2_-VASc scores were used to extract the Delta CHA_2_DS_2_-VASc score. The stroke predicting accuracies of the baseline, follow-up, and Delta CHA_2_DS_2_-VASc scores were assessed through regression analyses.

**Results:**

The mean baseline, follow-up, and Delta CHA_2_DS_2_-VASc scores were 4.2, 4.8, and 0.6 respectively. Ischemic stroke occurred in 54 (4.4%) patients, of which 83.3% had a Delta CHA_2_DS_2_-VASc score ≥1, contrary to 40.1% of the stroke-free group. The stroke risk per 1-point increase of the CHA_2_DS_2_-VASc score was not significantly associated with the baseline score (aHR=1.14; 95%CI: 0.93-1.41; *p*=0.201), whereas a significant association was observed with the follow-up (aHR=2.58; 95% CI: 2.07-3.21; *p*<0.001) and Delta (aHR=4.56; 95%CI: 3.50-5.94; *p*<0.001) scores. C-index assessment indicated that follow-up and Delta CHA_2_DS_2_-VASc scores were more potent predictors of ischemic stroke compared to baseline.

**Conclusion:**

In atrial fibrillation patients, changes in CHA_2_DS_2_-VASc score over time were associated with the incidence of stroke. The improved predictability of follow-up and Delta CHA_2_DS_2_-VASc scores indicates that stroke risk is not a static parameter.

**Trial registration:**

This is an observational, post-hoc analysis of the MISOAC-AF randomized controlled trial, registered on ClinicalTrials.gov (identifier: NCT02941978; registered: October 21, 2016).

**Supplementary Information:**

The online version contains supplementary material available at 10.1007/s00380-023-02278-1.

## Introduction

Atrial fibrillation (AF) dominates the field of cardiac arrhythmias by being the most common arrhythmia globally, with a rapidly increasing occurrence and an extensive disease burden [1, 2]. The paramount risk though for patients with AF remains the high incidence of stroke and other thrombotic events [3]. Thus, stroke risk assessment with CHA_2_DS_2_-VASc score [Congestive heart failure, Hypertension, Age ≥75 years (doubled), Diabetes mellitus, prior Stroke, transient ischemic attack, or systemic thromboembolism (doubled), Vascular disease, Age 65 to 74 years, Sex category (female)] is essential to AF management. By recommendation of recent guidelines, an elevated CHA_2_DS_2_-VASc score necessitates the initiation of oral anticoagulant (OAC) therapy as a protective strategy. Notably, despite acknowledging that the risk of stroke is dynamic, no specific recommendations are made regarding the frequency of reassessments [4, 5].

As the majority of AF patients are among the elderly population, they present with an inconstant variety of stroke risk-contributing comorbidities [6]. Yet, the evaluation of their outcomes has been conducted solely with the consideration of baseline risk factors in score validation studies [7].

In this study we aimed to assess the connection between the accumulation of new-onset comorbidities, depicted as changes in CHA_2_DS_2_-VASc score (i.e., Delta CHA_2_DS_2_-VASc score) over time, and the incidence of ischemic stroke in a population of AF patients receiving OAC treatment. The prognostic significance of the Delta CHA_2_DS_2_-VASc score has not been yet fully understood; therefore, we evaluated its relationship with ischemic stroke occurrence, as opposed to the baseline and follow-up score values.

## Materials and methods

### Study population

This study constitutes a post-hoc, observational analysis of the MISOAC-AF trial (Motivational Interviewing to Support Oral Anti Coagulation adherence in patients with non-valvular Atrial Fibrillation, ClinicalTrials.gov identifier: NCT02941978), a prospective randomized controlled trial, conducted in the cardiology ward of a tertiary hospital. The detailed study design and its results have been published previously [8, 9]. In essence, MISOAC-AF aimed to identify the effect of a specific physician-patient interview on a patient’s adherence to OAC therapy [9].

The baseline population of the present study consisted, in its entirety, of adult patients with non-valvular AF who were enrolled in MISOAC-AF. The participants were originally recruited between December 2015 and June 2018. Study approval was obtained by the Ethics Committee of the Aristotle University of Thessaloniki (Reference 173/30.11.2015). The study was in accordance with the principles of the declaration of Helsinki [10]. Patients provided their written, informed consent, prior to their participation.

### Data collection

All associated data, such as demographic and baseline clinical characteristics, personal information, medication history and baseline CHA_2_DS_2_-VASc score were obtained from the database of the MISOAC-AF trial. For the present study, the patients were subsequently followed-up annually via telephone interview until February 2020. The interview consisted of questions regarding any acquired comorbidities and ischemic stroke incidents that were previously confirmed by their corresponding physicians. Patients that could not be contacted for the follow-up surveys were excluded from further analyses. Moreover, after their written consent, the patients’ electronic healthcare records were accessed through the Greek national prescription registry, to collect information regarding medication history and co-existing conditions and verify the occurrence of all deaths. The registry contains information regarding prescription protocols and the medical conditions (including an ICD-10 designation) for which they were prescribed. Additionally, all deaths are by law registered on the platform.

### Data processing and statistical analysis

The investigated outcome was the incidence of ischemic stroke. The electronic records were used in conjunction with the details provided during the follow-up interviews, to determine stroke events and the onset of any new comorbidities. All the above data were used to calculate the participants’ follow-up CHA_2_DS_2_-VASc score, which was defined as the cumulative score at the end of the follow-up period or at the time of death. Changes in the CHA2DS2-VASc parameters were included in the calculation of the follow-up CHA2DS2-VASc score regardless of the time on which they were manifested during the follow-up period. The subtraction of the two scores (follow-up minus baseline score value) resulted in the Delta CHA_2_DS_2_-VASc score.

Following the calculation of the Delta CHA_2_DS_2_-VASc score, the patients’ data underwent statistical analyses. Continuous variables were summarized with means and standard deviations (SDs), while frequencies and percentages were used for categorical variables. The baseline and post-follow-up characteristics among participants whose Delta CHA_2_DS_2_-VASc score differed were compared with Students *t*-test or one-way analysis of variance (ANOVA) in the case of continuous variables, either using the Pearson chi-square test or Fisher’s exact test (whenever the expected count was less than 5) for categorical variables. Annual stroke event rates were calculated for each score of the three scoring systems (baseline, follow-up, and Delta CHA_2_DS_2_-VASc scores). The association between the different baseline, follow-up, and Delta CHA_2_DS_2_-VASc scores, and the incidence of ischemic stroke during the follow-up period was assessed with univariate and multivariate Cox proportional hazards models. The time-to-stroke variable used for these analyses was recorded as the number of days between the starting date of the patient’s follow-up and the event. The multivariate models included the relevant CHA_2_DS_2_-VASc score, baseline age, gender, prescription for OAC (vitamin K antagonist or direct oral anticoagulant), adherence to OAC treatment (defined as a proportion of days covered >80%, as recommended by the Pharmacy Quality Alliance [11]) and history of prior ischemic stroke as covariates. The prognostic value of the baseline, follow-up and Delta CHA_2_DS_2_-VASc score was examined by calculating C-indices, using the area under the receiver-operating characteristic (ROC) curve (AUC). The comparison of these values was performed using DeLong’s test. Kaplan–Meier survival analysis was performed to investigate the time-to-stroke data, according to different values of Delta CHA_2_DS_2_-VASc score. Comparisons between groups were possible with the log-rank test. All results are reported with the corresponding 95% confidence interval (CI). A *p*-value less than 0.05 was considered statistically significant. Analyses were performed using IBM SPSS statistics version 28.0.1.1 (International Business Machines Corporation, New York, United States of America) and MedCalc version 20.114 (MedCalc Software Ltd, Ostend, Belgium).

## Results

### Baseline and follow-up characteristics

The study cohort consisted of 1127 patients, out of 1140 initially recruited in MISOAC-AF; 13 participants could not be contacted for follow-up. Female individuals constituted 45.3% of the sample. The most common comorbidities of the study population at baseline were arterial hypertension (79.1%) and congestive heart failure (CHF) (49.2%). The baseline and post-follow-up characteristics, stratified by the Delta CHA_2_DS_2_-VASc score, are provided in Table 1. The median time interval between enrollment and the last date of follow-up was 2.6 years. The mean patients’ age increased from 73.6 to 76.6 years in the time of follow-up. Similarly, mean CHA_2_DS_2_-VASc score were 4.2 at baseline and rose to 4.8 at follow-up.

**Table 1:** Baseline and follow-up characteristics of patients with atrial fibrillation stratified by Delta CHA_2_DS_2_-VASc score

		Delta CHA_2_DS_2_-VASc^a^ score				
	All patients (n=1127)	Score 0	Score 1	Score 2	Score ≥3	*p*-value
		652 (57.9%)	354 (31.4%)	95 (8.4%)	26 (2.3%)	
Baseline characteristics^b^						
Age, years	73.6 ± 10.9	75.6 ± 11.5	70.1 ± 9.6	73.0 ± 8.2	72.2 ± 7.1	<0.001
Age 65-74 years	289 (25.6%)	78 (12.0%)	158 (44.6%)	39 (41,1%)	14 (58.3%)	<0.001
Age ≥ 75 years	614 (54.5%)	482 (73,9%)	89 (25.71%)	35 (36.8%)	8 (30.8%)	<0.001
Female gender	511 (45.3%)	301 (46.2%)	151 (42.7%)	49 (51.6%)	10 (38.5%)	0.362
BMI^c^ (Kg/m^2^)	28.5 ± 5.4	28.3 ± 5.5	29.1 ± 5.4	28.1 ± 5.0	27.9 ± 5.0	0.171
Coronary artery disease	440 (39.0%)	264 (40.5%)	134 (37.9%)	32 (33.7%)	10 (38.5%)	0.586
Chronic kidney disease	473 (42.0%)	328 (50.3%)	107 (30.2%)	32 (33.7%)	6 (23.1%)	<0.001
Congestive heart failure	555 (49.2%)	388 (59.5)	132 (37.3%)	25 (26.3%)	10 (38.5%)	<0.001
Hypertension	892 (79.1%)	536 (82.2%)	271 (76.6%)	70 (73.7%)	15 (57.7%)	0.003
Diabetes mellitus	382 (33.9%)	231 (35.4%)	117 (33.1%)	29 (30.5%)	5 (19.2%)	0.287
Vascular diseases	507 (45.0%)	328 (50.3%)	140 (39.5%)	29 (30.5%)	10 (38.5%)	<0.001
Persistent or permanent AF^d^	572 (50.8%)	349 (53.5%)	164 (46.3%)	48 (50.5%)	11 (42.3%)	0.137
CHA_2_DS_2_-VASc score	4.2 ± 1.9	4.7 ± 1.9	3.5 ± 1.6	3.4 ± 1.5	3.1 ± 1.8	<0.001
Prescription for VKA^e^	330 (29.3%)	200 (30.7%)	95 (26.8%)	25 (26.3%)	10 (38.5%)	0.377
Prescription for DOAC^f^	611 (54.2%)	344 (52.8%)	198 (55.9%)	56 (58.9%)	13 (50.0%)	0.568
Follow-up characteristics^b^						
Age, years	76.6 ± 10.7	78.5 ± 11.3	73.2 ± 9.5	76.2 ± 8.0	75.7 ± 6.9	<0.001
Age 65-74 years	199 (17.7%)	78 (12.0%)	97 (27.4%)	19 (20.0%)	5 (19.2%)	<0.001
Age ≥ 75 years	797 (70.7%)	482 (73.9%)	224 (63.3%)	71 (74.7%)	20 (76.9%)	0.003
CHA_2_DS_2_-VASc score	4.8 ± 1.8	4.7 ± 1.9	4.5 ± 1.6	5.4 ± 1.5	6.2 ± 1.7	<0.001
Prescription for VKA	286 (25.4%)	179 (27.5%)	79 (22.3%)	21 (22.1%)	7 (26.9%)	0.283
Prescription for DOAC	696 (61.8%)	382 (58.6%)	232 (65.5%)	65 (68.4%)	17 (65.4%)	0.077
Patient adherent to OAC^g^ treatment	623 (55.3%)	369 (56.6%)	191 (54.0%)	48 (50.5%)	15 (57.7%)	0.651
Events during follow-up^b^						
Stroke	54 (4.4%)	9 (1.4%)	5 (1.4%)	26 (27.4%)	14 (53.8%)	<0.001
Cardiovascular death	312 (27.7%)	206 (31.6%)	79 (22.3%)	21 (22.1%)	6 (23.1%)	0.008
All-cause death	422 (47.4%)	288 (44.2%)	103 (29.1%)	25 (26.3%)	6 (23.1%)	<0.001

^a^CHA_2_DS_2_-VASc score = congestive heart failure, hypertension, age ≥ 75 years, diabetes mellitus, prior stroke, vascular disease, age 65-74 years, sex category (female)

^b^Values are mean ± standard deviation or n (%)

^c^BMI = body mass index

^d^AF = atrial fibrillation

^e^VKA = vitamin K antagonist

^f^DOAC = direct oral anticoagulant

^g^OAC = oral anticoagulant

**Fig. 1:**
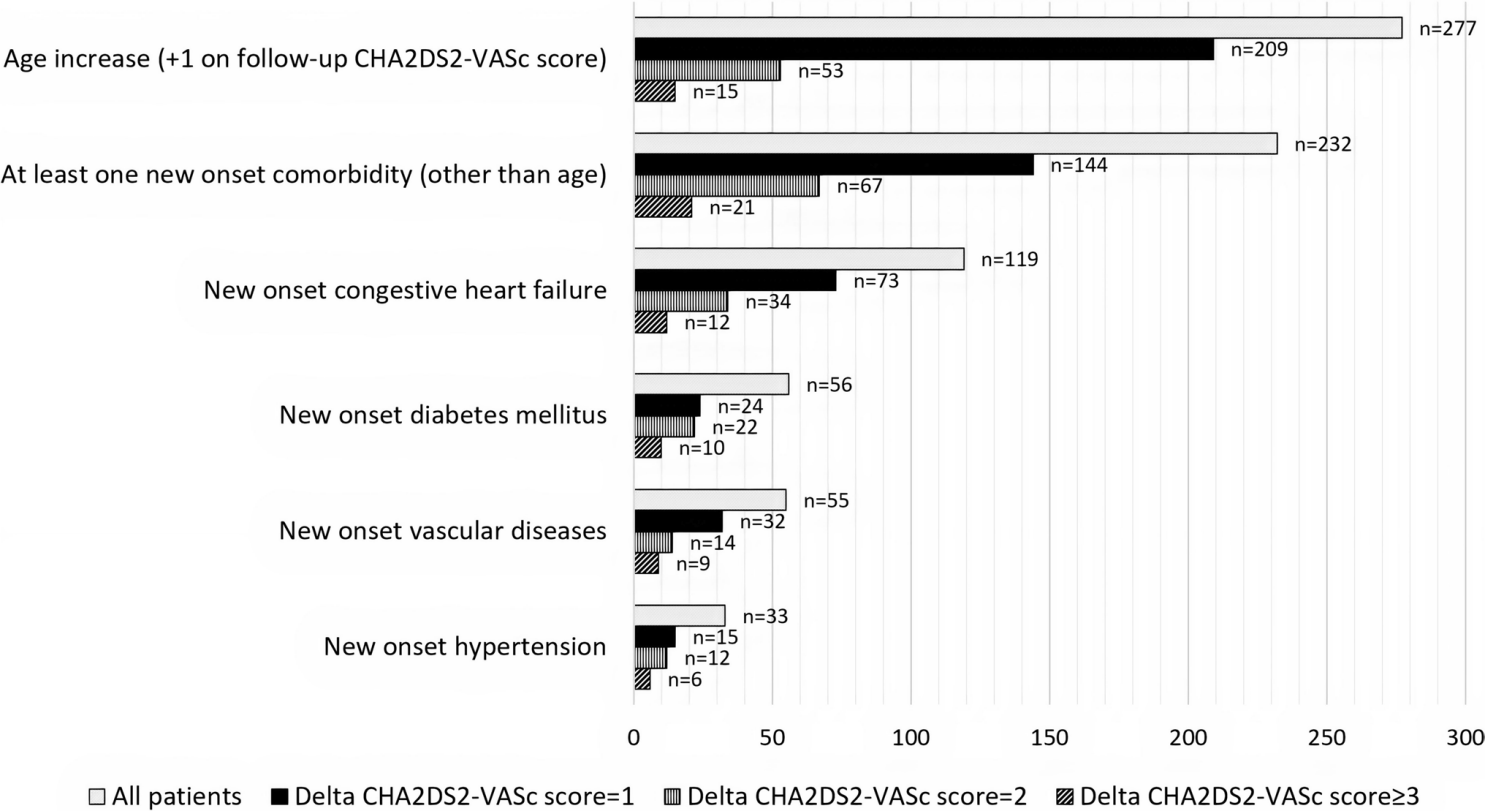
Contributing factors to the increase of the CHA_2_DS_2_-VASc score during the follow-up period

A considerable proportion of the research sample (42.1%) acquired additional CHA_2_DS_2_-VASc score-related comorbidities or became older than 65 or 75 years old throughout the follow-up period. The development of at least one novel comorbidity other than increasing age concerned 232 (20.6%) participants. The most commonly occurring was CHF (10.6%), followed by diabetes mellitus (5.0%). Detailed information regarding the new-onset comorbidities is provided in Fig. 1 and Table 1.

With regards to CHF, a higher number of patients with a positive baseline history had persistent or permanent AF (*n*=358, 64.5%), compared to patients without CHF (*n*=214, 37.4%; *p*<0.001). However, there was no statistically significant difference between the number of patients with confirmed persistent or permanent AF that developed CHF during follow-up (*n*=53, 9.3%), compared to patients with paroxysmal AF (*n*=66, 11.9%; *p*=0.152).

### Risk of ischemic stroke and influence of the Delta CHA_2_DS_2_-VASc score

**Fig. 2:**
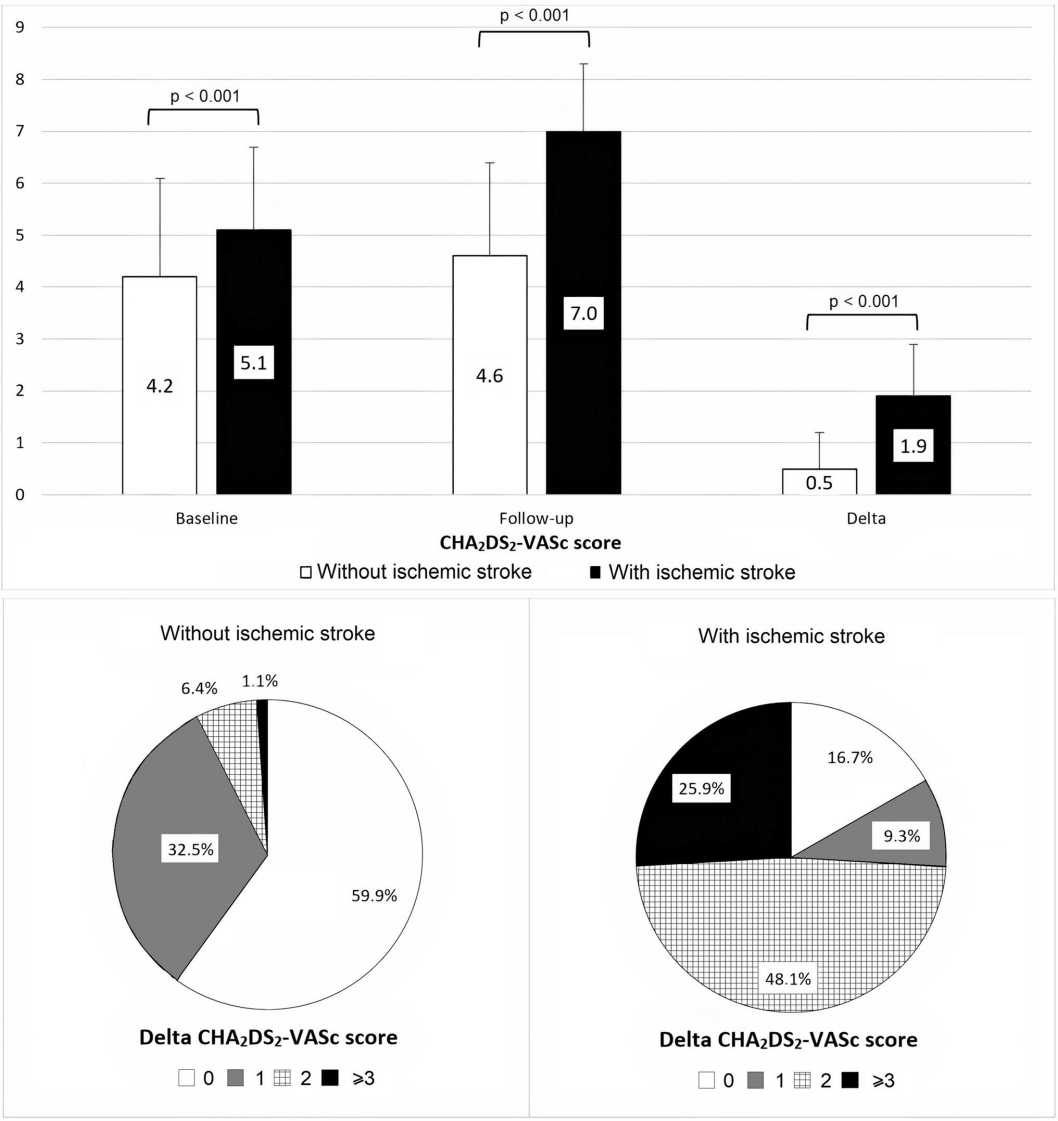
Baseline, Follow-up, and Delta CHA_2_DS_2_-VASc scores of patients with or without ischemic stroke during the follow-up period

**Fig. 3:**
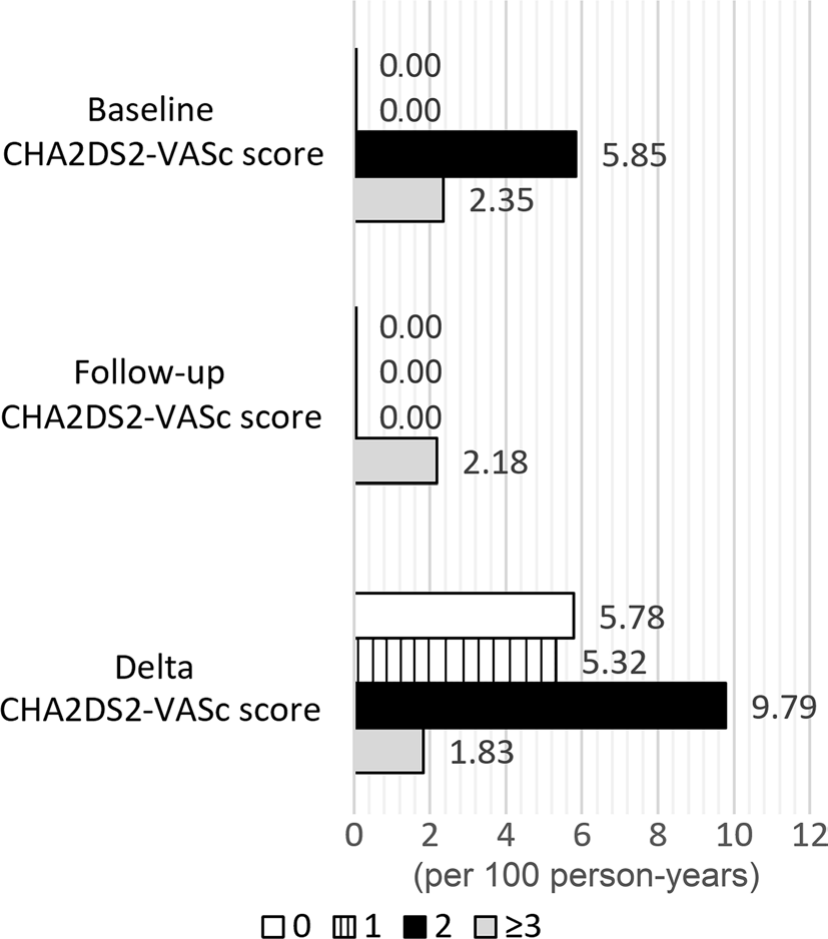
Annual stroke event rates per score of the Baseline, Follow-up, and Delta CHA_2_DS_2_-VASc scores

The outcome of ischemic stroke occurred in 54 (4.4%) participants during follow-up. The baseline, follow-up, and Delta CHA_2_DS_2_-VASc scores of these patients were significantly higher compared to ones who did not experience the event (Fig. 2). Accordingly, the majority of patients with stroke during follow-up (*n*=45, 83.3%) had a Delta CHA_2_DS_2_-VASc score ≥ 1, whereas the corresponding percentage of event-free patients was only 40.1% (Fig. 2). The annual stroke event rates for each score of the three scoring systems are shown on Fig. 3.

Univariate and multivariate Cox regression analysis models are demonstrated on Table 2 and Supplemental Table S1. The multivariate model indicated that the baseline CHA_2_DS_2_-VASc score [adjusted Hazard Ratio (aHR) =1.14; 95%CI: 0.93–1.41; *p*=0.201] was not significantly correlated with the risk of ischemic stroke during follow-up. However, the follow-up (aHR = 2.58; 95%CI: 2.07–3.21; *p*<0.001) and Delta CHA_2_DS_2_-VASc scores (aHR=4.56; 95%CI: 3.50-5.94; *p*<0.001) were independently associated with increased risk of ischemic stroke. Notably, the prescription for OAC (vitamin K antagonist or direct oral anticoagulant) and patients’ adherence to OAC treatment were not significantly associated with reduced risk of stroke in either of the above models.

**Table 2:** Risk of ischemic stroke based on Baseline, Follow-up, and Delta CHA_2_DS_2_-VASc score

	Hazard Ratio	95% CI^a^	*p*-value
Univariate models			
Baseline CHA_2_DS_2_-VASc^b^ score	1.35	1.17 – 1.55	<0.001
Follow-up CHA_2_DS_2_-VASc score	2.19	1.85 – 2.59	<0.001
Delta CHA_2_DS_2_-VASc score	3.47	2.77 – 4.34	<0.001
Multivariate models (covariates: baseline age, gender, prescription for OAC^c^, adherence to OAC treatment, history of prior ischemic stroke)			
Baseline CHA_2_DS_2_-VASc score	1.14	0.93 – 1.41	0.201
Follow-up CHA_2_DS_2_-VASc score	2.58	2.07 – 3.21	<0.001
Delta CHA_2_DS_2_-VASc score	4.56	3.50 – 5.94	<0.001

^a^95% CI = 95% confidence interval

^b^CHA_2_DS_2_-VASc score = congestive heart failure, hypertension, age ≥ 75 years, diabetes mellitus, prior stroke, vascular disease, age 65-74 years, sex category (female)

^c^OAC = oral anticoagulant

**Fig. 4:**
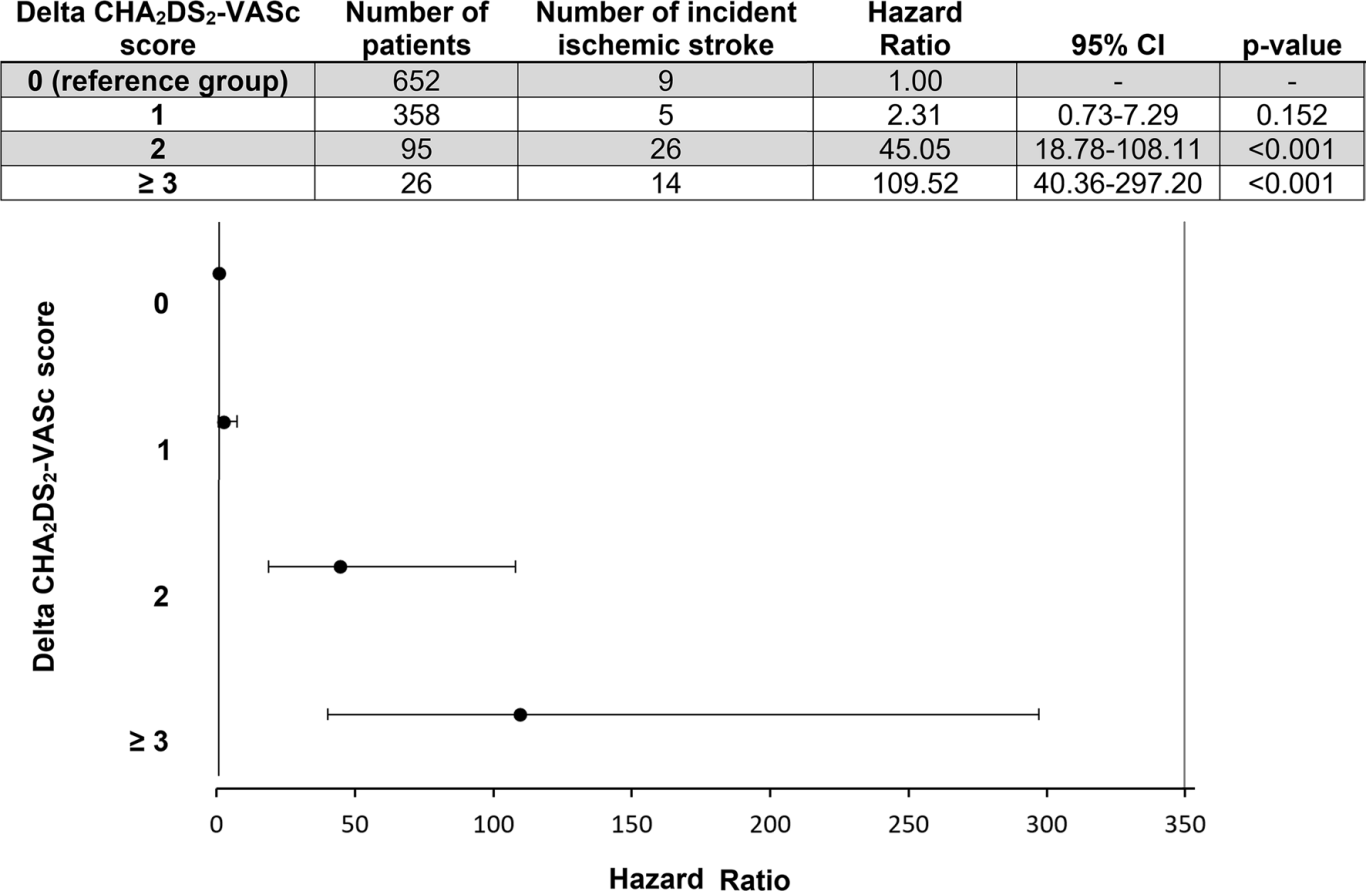
Risk of ischemic stroke stratified by Delta CHA_2_DS_2_-VASc score (adjusted for baseline age, gender, prescription for OAC, adherence to OAC treatment and prior history of ischemic stroke)

The individual effect of specific ranges of Delta CHA_2_DS_2_-VASc score on the risk for ischemic stroke throughout the follow-up was assessed with Delta CHA_2_DS_2_-VASc score 0 as the reference group. Among them, Delta CHA_2_DS_2_-VASc score ≥3 constituted the most pronounced risk factor for the occurrence of stroke, with an adjusted HR of 109.52 (95%CI: 40.36–297.20; *p*<0.001) (Fig. 4).

### Impact of the Delta CHA_2_DS_2_-VASc score on stroke-free survival time

**Fig. 5:**
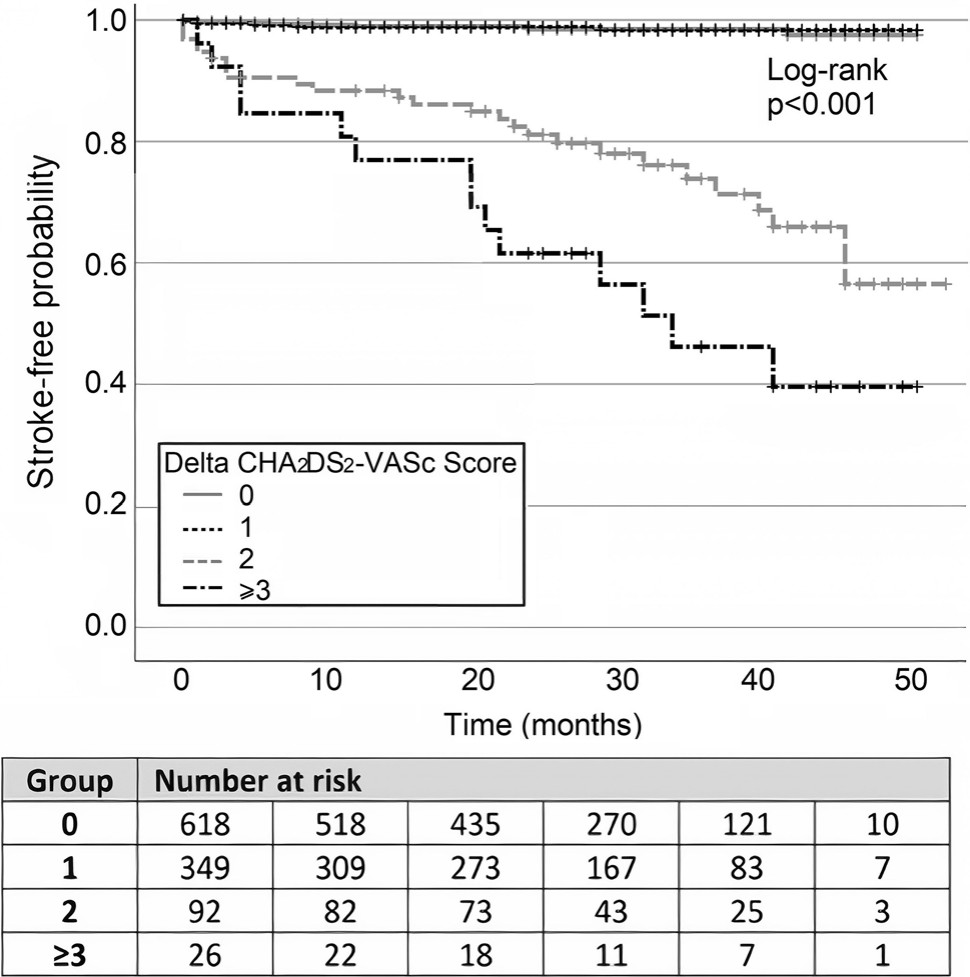
Kaplan – Meier analysis survival curves for separate groups of Delta CHA_2_DS_2_-VASc score and the occurrence of ischemic stroke

The Kaplan–Meier curve for the stroke-free probability, for different Delta CHA_2_DS_2_-VASc score values, is illustrated in Fig. 5. The estimated 2-year stroke-free probability was 98.5%, 98.8%, 81.1%, and 61.5% for patients with a Delta CHA_2_DS_2_-VASc score of 0, 1, 2, and ≥3 respectively. Correspondingly, the 4-year stroke-free probability was 97.5%, 98.3%, 56.5%, and 39.6% for patients with a Delta CHA_2_DS_2_-VASc score of 0, 1, 2, and ≥3 respectively (both *p*<0.001).

### Predictive validity of the Delta CHA_2_DS_2_-VASc score on ischemic stroke incidence

**Fig. 6:**
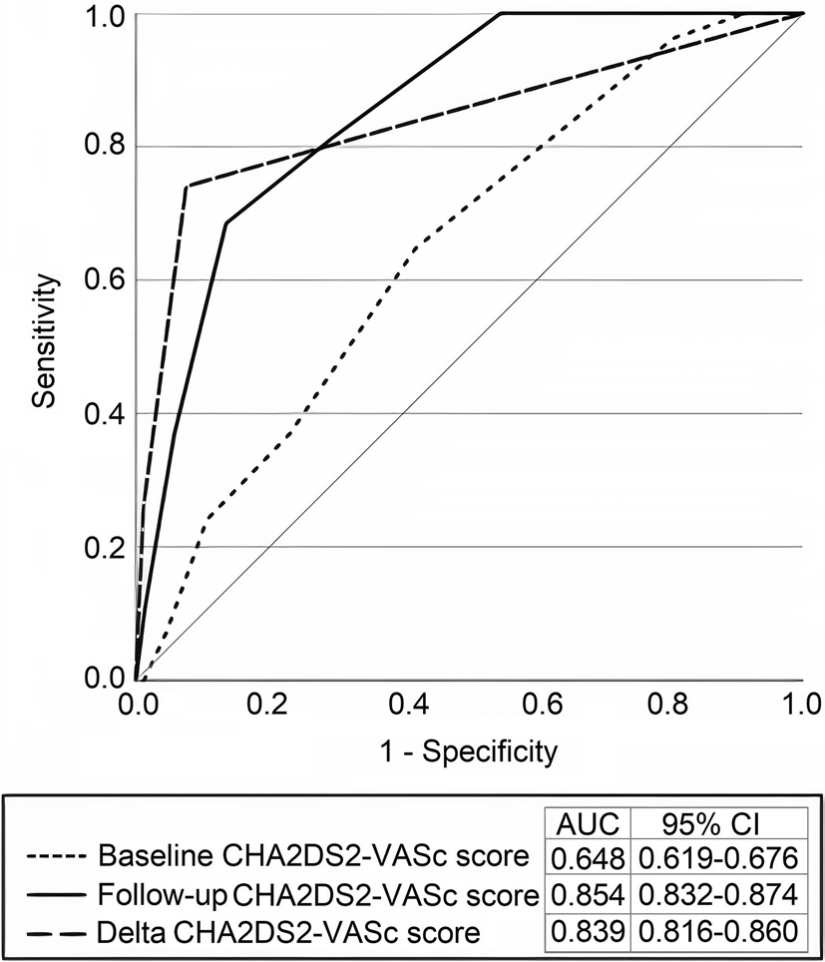
Receiver operating characteristics (ROC) curve for the Baseline, Follow-up, and Delta CHA_2_DS_2_-VASc scores in predicting ischemic stroke

The ROC curve for the baseline, follow-up, and Delta CHA_2_DS_2_-VASc scores, and the occurrence of stroke events was created (Fig. 6). The AUC for the follow-up (0.854; 95%CI: 0.832–0.874; *p*<0.001) and Delta (0.839; 95%CI: 0.816–0.860; *p*=0.002) CHA_2_DS_2_-VASc scores were significantly higher in contrast to the baseline score (0.648; 95%CI: 0.619–0.676). Delta CHA_2_DS_2_-VASc score’s predicting value was not found superior to that of the follow-up score (*p*=0.723).

## Discussion

This observational study indicates the dynamic state of stroke risk in patients with AF, as assessed through the CHA_2_DS_2_-VASc score. It was found that a substantial proportion (20.6%) of our study cohort developed at least one new comorbidity, other than increasing age, during follow-up. It was also revealed that the majority (83.3%) of patients who experienced an ischemic stroke in the course of follow-up had at least one point increase in their CHA_2_DS_2_-VASc score (Delta CHA_2_DS_2_-VASc score ≥1). Correspondingly, a greater Delta CHA_2_DS_2_-VASc score was indicative of higher risk of stroke occurrence.

The predictive value of Delta CHA_2_DS_2_-VASc score was firstly assessed in a sample of 31,039 patients with AF, by Chao et al. [12]. The investigated cohort consisted exclusively of patients without a prescription for OACs or any CHA_2_DS_2_-VASc score-related comorbidities, other than age and gender. Among them, the ones that experienced an ischemic stroke during follow-up had a significantly higher Delta CHA_2_DS_2_-VASc score (1.86 vs 0.89, *p*<0.001). The authors also revealed the association of higher Delta CHA_2_DS_2_-VASc scores with a higher risk of stroke, whereas it also performed better as a stroke predictor, when compared to baseline or follow-up CHA_2_DS_2_-VASc scores. Our study’s findings confirm these associations, except for the better performance of Delta CHA_2_DS_2_-VASc score in predicting the occurrence of stroke, compared to the follow-up score. However, in our study, follow-up as well as Delta CHA_2_DS_2_-VASc scores performed significantly better than the baseline score. This finding combined with the not significant correlation of the baseline score with stroke risk, is not in agreement with the guidelines’ recommendation to use the CHA_2_DS_2_-VASc score for the assessment of stroke risk [4, 5]. It should be noted though that this is a real-world observational study and non-recognized confounders might have influenced our adjusted analyses. Regardless, our results support the non-static nature of stroke risk in AF and the validity of the CHA_2_DS_2_-VASc score as a reliable tool for reassessment.

The Delta CHA_2_DS_2_-VASc score was further evaluated in a retrospective study which included roughly 160,000 AF patients with CHA_2_DS_2_-VASc score-related comorbidities, although those receiving OACs at baseline were excluded [13]. A significantly higher risk of stroke was associated with the reclassification of a patient to a higher CHA_2_DS_2_-VASc score category, during a 10-year follow-up. Additionally, in agreement with our findings, the most recently calculated CHA_2_DS_2_-VASc score and Delta CHA_2_DS_2_-VASc score, held a superior predictive role in prognosticating stroke occurrence.

A more recent study appraised the association of Delta CHA_2_DS_2_-VASc score and the prevalence of stroke, in more than 600,000 individuals [14]. It was demonstrated that the majority (67.1%) of patients who experienced an ischemic stroke had a Delta CHA_2_DS_2_-VASc score ≥1. Distinctly, both follow-up and Delta CHA_2_DS_2_-VASc scores performed better than the baseline score, in outcome predicting, with a slight lead of the follow-up score, which is corroborated by our study.

Evidently, the previous studies enforced strict enrollment criteria to their participants. With the exception of the study by Yoon et al. [13], the remaining researchers excluded patients with baseline comorbidities from their investigations [12, 14]. Patients on OAC therapy were notably excluded from all prior analyses. Nevertheless, it is acknowledged that the majority of patients suffering from AF present with several comorbidities and the recipients of OAC treatment account for almost 80% [15, 16]. Hence, our study aimed to include a more representative sample of patients with non-valvular AF, as they are encountered under real-world circumstances. The involvement of patients with various prior risk-contributing comorbidities, indicated that whatever the previous co-existing conditions, the accumulation of additional ones was associated with an increase in the overall risk of stroke, even when on OAC treatment. These conclusions, however, do not deviate from the preceding ones, while applying to a wider range of patients. The lack of correlation between the risk of stroke and prescription for and adherence to OAC treatment in our study can be explained by the presence of the aforementioned comorbidities, as well as the fact that OAC is a risk-reducing strategy that does not completely eliminate stroke risk [5].

Interestingly, despite the known association of the sustained types of AF with a higher incidence of CHF [17], in our cohort, new-onset CHF was not significantly associated with a history of persistent or permanent AF. This finding can be attributed to the high prevalence of pre-existing CHF with comorbid persistent or permanent AF at baseline.

Based on the results of our study, future research should assess the effectiveness of an increased vigilance management strategy for patients with higher-than-baseline follow-up CHA_2_DS_2_-VASc scores, with regular reassessments, detailed explanation of the risk for and signs of stroke and maximized efforts to control the associated comorbidities. Furthermore, our primary aim was not the proposition of a novel assessment tool for AF patients, based on the Delta CHA_2_DS_2_-VASc score, rather than to highlight the derivation of AF-associated stroke risk from the development of new comorbidities. It is, therefore, essential that stroke risk is reassessed frequently, regardless of the patient’s OAC status, since an updated CHA_2_DS_2_-VASc score presents a more reliable risk indicator.

### Limitations of the study

The observational nature of the study may involve limitations. The relatively small study sample may be a potential liability compared to previously performed research. However, the less restrictive enrollment criteria allowed a more adequate representation of real conditions. The determination of only two CHA_2_DS_2_-VASc scores – baseline and follow-up – without the inclusion of intermediate score values may also pose a limitation. Nonetheless, the follow-up CHA_2_DS_2_-VASc score was considered as indicative of the entirety of the patients’ prior and acquired comorbidities. A variety of patient data were provided by the patients themselves. Even though that might had involved limitations, the information regarding their prescribed medication, comorbidities, and mortality was cross-referenced as to its validity through the Greek national prescription registry. Moreover, the low sample size in the higher delta CHA_2_DS_2_-VASc score subgroups has resulted in wide confidence intervals in the survival analysis. Despite their statistical significance, results should be interpreted with caution with regards to the stroke risk associated with individual delta score values. The inclusion of participants solely of Greek origin may have resulted in reduced applicability of the findings to other races.

## Conclusions

The stroke risk in AF patients is non-static, as demonstrated by the changes in their CHA_2_DS_2_-VASc score over time. The vast majority of patients with AF, who developed an ischemic stroke during the period of their follow-up had a concurrent presentation of new-onset comorbidities. Concomitantly, both follow-up and Delta CHA_2_DS_2_-VASc scores performed better in predicting stroke incidents, which indicates the dynamic nature of stroke risk as patients age and acquire additional risk factors. This signifies the importance of regular and thorough reassessments of patients with AF.

## Supplementary Information

Below is the link to the electronic supplementary material.

Supplementary file1 (PDF 59 KB)

## Data Availability

Anonymized data can be made available upon reasonable request to the corresponding author.
