# Supplementary material for: Changes in CHA2DS2-VASc score and risk of ischemic stroke among patients with atrial fibrillation
Source: Heart Vessels. 2023 Jun 13;38(10):1267–76. doi: 10.1007/s00380-023-02278-1 (PMC10465382; doi:10.1007/s00380-023-02278-1)
Supplement: Supplementary file 1 — Supplementary file1 (PDF 59 KB) [file 380_2023_2278_MOESM1_ESM.pdf]

Article title:

Changes in CHA_2DS_2-VASc score and risk of ischemic stroke among patients with atrial fibrillation

Journal name:

Heart and Vessels

Authors:

Eirinaios Tsiartas[1], Athanasios Samaras[1], Andreas S. Papazoglou[1], Anastasios Kartas[1], Dimitrios V. Moysidis[1], Eleftherios Gemousakakis[1], Odysseas Kamzolas[1], Alexandra Bekiaridou[1], Ioannis Doundoulakis[1], Apostolos Tzikas[1], George Giannakoulas[1]

[1]First Cardiology Department, AHEPA University Hospital, School of Medicine, Faculty of Health Sciences, Aristotle University of Thessaloniki, Thessaloniki, Greece

Corresponding author:

George Giannakoulas, MD, PhD, Associate Professor of Cardiology

First Cardiology Department, AHEPA University Hospital,

School of Medicine, Faculty of Health Sciences, Aristotle University of Thessaloniki,

Thessaloniki, Greece

Email: g.giannakoulas@gmail.com /

Supplemental Table S1: Multivariate Cox-regression models for the risk of ischemic stroke based on Baseline, Follow-up, and Delta CHA_2DS_2-VASc score

	**Hazard Ratio**	**95% CI[a]**	**p-value**
**Multivariate model for Baseline CHA_2DS_2-VASc[b] score**			
Baseline CHA_2DS_2-VASc score	1.14	0.93-1.41	0.201
Age	1.02	0.99-1.06	0.219
Gender (female)	1.55	0.86-2.78	0.146
Prescription for OAC[c]			
- VKA[d]	1.49	0.64-3.48	0.356
- DOAC[e]	0.72	0.31-1.68	0.442
Adherence to OAC treatment	0.88	0.52-1.51	0.652
History of prior ischemic stroke	1.61	0.68-3.82	0.279
**Multivariate model for Follow-up CHA_2DS_2-VASc score**			
Follow-up CHA_2DS_2-VASc score	2.58	2.07-3.21	<0.001
Age	0.98	0.95-1.02	0.371
Gender (female)	0.64	0.35-1.20	0.163
Prescription for OAC			
- VKA	1.04	0.44-2.44	0.935
- DOAC	0.63	0.27-1.49	0.292
Adherence to OAC treatment	0.83	0.48-1.43	0.505

History of prior ischemic stroke	0.38	0.18-0.79	0.010
**Multivariate model for Delta CHA_2DS_2-VASc score**			
Delta CHA_2DS_2-VASc score	4.56	3.50-5.94	<0.001
Age	1.07	1.03-1.11	<0.001
Gender (female)	2.40	1.30-4.44	0.005
Prescription for OAC<br>- VKA<br>- DOAC	 1.36<br>0.703	 0.58-3.19<br>0.29-1.68	 0.480<br>0.427
Adherence to OAC treatment	0.97	0.55-1.68	0.901
History of prior ischemic stroke	8.41	3.89-18.20	<0.001

[a]CI = confidence interval

[b]CHA_2DS_2-VASc score = congestive heart failure, hypertension, age ≥ 75 years, diabetes mellitus, prior stroke, vascular disease, age 65-74 years, sex category (female)

[c]OAC = oral anticoagulant

[d]VKA = vitamin K antagonist

[e]DOAC = direct oral anticoagulant
